# The effect of midwife-oriented breastfeeding counseling on self-efficacy and performance of adolescent mothers: a clinical trial study

**DOI:** 10.1186/s12884-023-05982-y

**Published:** 2023-09-19

**Authors:** Sepideh Hosseinzadeh Fahim, Farideh Kazemi, Sayedeh Zahra Masoumi, Mansoureh Refaei

**Affiliations:** 1 Department of Mother and Child Health, School of Nursing and Midwifery, Hamadan University of Medical Sciences, Hamadan, Iran; 2 Department of Midwifery and Reproductive Health, School of Nursing and Midwifery, Hamadan University of Medical Sciences, Hamadan, Iran; 3 Department of Mother and Child Health, Mother and Child Care Research Center, School of Nursing and Midwifery, Hamadan University of Medical Sciences, Hamadan, Iran

**Keywords:** Breast feeding, Counseling, Self-efficacy, Adolescent

## Abstract

**Background:**

Breastfeeding behaviors are strongly influenced by self-efficacy. This research aimed to determine the effect of breastfeeding counseling based on the Ready Set Baby (RSB) education program on self-efficacy and breastfeeding performance in adolescent mothers.

**Methods:**

In 2022, a parallel randomized clinical trial was carried out in Hamadan city's comprehensive health centers, involving 64 pregnant teenagers. The block randomization method was employed to divide the participants into two groups. The data collection instruments were a demographic characteristics questionnaire, a breastfeeding self-efficacy questionnaire, and the Bristol breastfeeding checklist. Three individual counseling sessions during pregnancy were conducted based on the "RSB" program. The ANCOVA was used for comparing groups. The statistical analyst was blinded to the group assignment.

**Results:**

The study included 64 participants with a mean age of 16.97(1.30) years, data from 60 participants were analyzed. The demographic and clinical characteristics of the two groups were relatively similar (*P* > 0.05). Following the intervention, self-efficacy and breastfeeding performance scores were measured and adjusted for confounding factors. The mean scores for self-efficacy were 116.03(20.64) and 100.02(20.64) (*P* < 0.005), with effect size 0.77 [MD = 16.01 (95% CI: 5.34,26.67)], and the mean scores for breastfeeding performance were 6.30(2.07) and 4.12(2.07) (*P* < 0.002), with effect size 1.05 [MD = 2.18 (95% CI: 1.11,3.24)] in the intervention and control groups, respectively.

**Conclusions:**

The Ready Set Baby education program's breastfeeding counseling for primiparous adolescent pregnant women significantly boosted their self-efficacy and performance in breastfeeding. Given the crucial role of breastfeeding in ensuring the well-being of both mother and child, further research is imperative to identify suitable and impactful interventions that can encourage breastfeeding practices among adolescents.

**Trial registration:**

The trial protocol of this study has been registered in Iranian Registry of Clinical Trials at 08/09/2021. The registration reference is IRCT20200530047596N3.

## Background

Adolescent pregnancy poses a significant challenge in numerous regions across the globe [1], resulting in adverse health and socioeconomic outcomes for the individual, family, and society. According to the World Health Organization, adolescence spans from 10 to 19 years old [2]. In countries with low or middle-income economies, approximately 21 million pregnancies annually occur among individuals aged 15 to 19 in 2019. Adolescent mothers face risks such as premature birth, underweight newborn, and critical neonatal states during and after pregnancy [3].

Annually, over 820,000 children are saved through the consumption of human milk, resulting in savings of over 300 billion dollars [4]. The World Health Assembly has set a goal of achieving an exclusive breastfeeding rate of at least 50% for infants under 6 months by 2025. However, between 2010 and 2018, this rate was only 45.7% in 57 low-income or middle-income economies [5]. Adolescent mothers have been found to have lower breastfeeding rates than older mothers [6]. Therefore, they require additional support [7]. Breastfeeding self-efficacy (BSE), a mother's confidence in her ability to breastfeed her newborn [8], is a critical factor in exclusive breastfeeding and breastfeeding duration [9]. Unfortunately, many first-time breastfeeding mothers lack the necessary knowledge and self-efficacy [10], particularly adolescent mothers who have been found to have lower BSE than other age groups [11]. A study showed that BSE is the primary factor affecting exclusive breastfeeding in adolescent mothers [12]. Educational and supportive interventions can effectively increase exclusive breastfeeding and BSE [8, 13]. Breastfeeding counseling by healthcare providers significantly contributes to breastfeeding performance [14]. Some literature has reported the beneficial impact of breastfeeding counseling and other interventions on continued exclusive breastfeeding, self-efficacy, and breastfeeding performance in non-teenage mothers [10, 15–17].

Studies have shown that implementing the Ready Set Baby (RSB) counseling approach, both individually and in groups, before birth has improved breastfeeding goals. RSB is a program designed to educate women on the benefits and proper handling of breastfeeding [18]. However, research has indicated a need to increase breastfeeding self-efficacy (BSE) in teenage mothers, which should be explored within midwifery practices [12]. The majority of studies on this topic have focused on non-teenage mothers.

As mentioned, improving self-efficacy has a positive effect on exclusive breastfeeding and breastfeeding duration. Improving the breastfeeding self-efficacy and performance of the mother, especially adolescent mothers, is effective on newborn outcomes, including saving the baby's life, as well as economic savings, so the authors of this article conducted this research intending to determine the effect of prenatal counseling based on the Ready Set Baby education program on self-efficacy and breastfeeding performance in adolescent mothers.

## Methods

This two-group randomized clinical trial study with a parallel design was conducted between September 2021 and November 2022.

### Participants

This study was conducted on nulliparous women under 19 years who received prenatal care in comprehensive health centers in Hamadan, in the west of Iran. The inclusion criteria were gestational age 30–33 weeks, a healthy singleton fetus, no high-risk pregnancy, literacy at sixth grade level (end of elementary school), living independently with her husband, and not suffering from physical and mental illnesses. Exclusion criteria were the presence of a known abnormality in the newborn or a problem in the mother's breast preventing breastfeeding, giving birth before week 37, and the occurrence of an unfortunate event (such as the newborn death, divorce, and loss of loved ones) during the study.

### Sampling

Based on the self-efficacy variable that was the primary outcome of this study, taking into account the alpha = 0.050, power = 0.90, m1 = 46.7, m2 = 52.9, sd1 = 6.5, sd2 = 7.0 and attrition rate of 25%, using the Sampsi module in Stata-13 software, the number of samples was determined to be 32 people in each group [19]. The randomization sequence was determined by one of the researchers, the student's supervisor, using permutation blocks of 4 by Random allocation software. After determining the sequence, 64 opaque envelopes were prepared in which the determined sequence was placed and numbered from 1 to 64. During the sampling, the envelopes were opened in order of number and the person was assigned to the designated group.Therefore, the researcher who did the sampling was not aware of the order of the sequence.

**Fig. 1:**
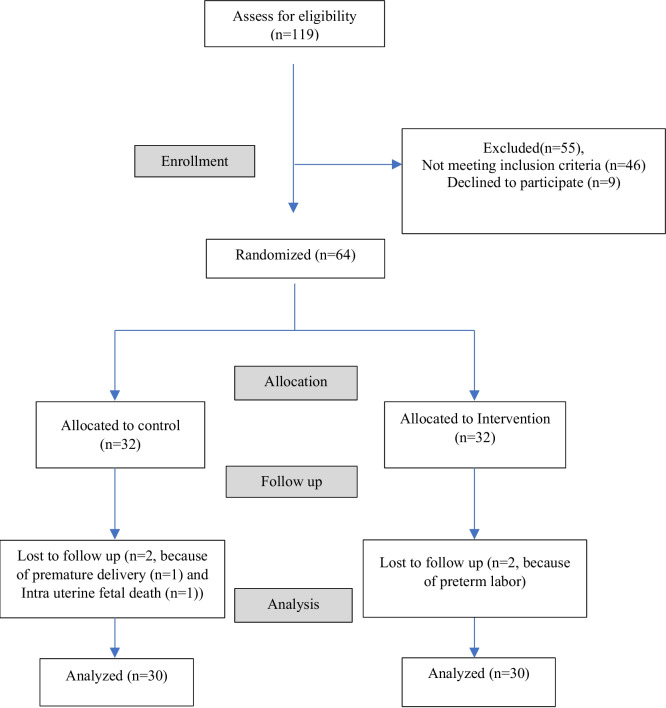
CONSORT flow diagram

The sampling setting was all comprehensive health centers of Hamedan City (36 centers). All pregnant women in the city are covered by these 36 centers. Using the Integrated Health Software (Sib), the researcher identified adolescent pregnant mothers (between 0–5 cases were identified in each center). A total of 119 mothers were identified, and after checking the inclusion criteria, 64 mothers were included in the study. The participants were then randomly assigned to either the intervention or control group using numbered envelopes. Four individuals were excluded from the study, leaving a sample size of 60 for analysis (3 people due to premature birth and one person due to intrauterine death). Notably, the statistical analyst was blinded to the group allocation (Fig. 1).

### Intervention and data collection method

After identifying the eligible people, in the 30th week of pregnancy, one of the researchers contacted the mother and explained the objectives of the study. The research sample was present at the comprehensive health center in the 30–33 week of pregnancy (in accordance with routine pregnancy care). At the time of registration of this trial, the following times were determined for intervention (the first session in weeks 31 to 34—the second session in weeks 35–37—the third session in weeks 38 to 40). Before the start of the intervention, the researchers concluded that some mothers may give birth at 38 weeks (before they received the third counseling session). Therefore, the time of the third session was changed to week 36–38, according to which, the time of the first and second session was also changed.

After that, the participants were assigned to the intervention or control group according to the envelope number. They were then asked to complete a demographic-clinical questionnaire and the breastfeeding self-efficacy scale (BSES). If the person was in the intervention group, the first counseling session was conducted by a researcher who was a master's student in midwifery counseling with ample experience in the delivery unit and breastfeeding counseling; then arrangements were made to participate in the next sessions. she provided three individual, face-to-face counseling sessions lasting 60–90 min in one of the rooms of the comprehensive health center. Before each session, the participants were contacted by phone to coordinate the next session, and if they had any questions, the researcher answered them. In total, each person was contacted twice.

The counseling was based on the Ready Set Baby curriculum, which includes a 28-page color booklet divided into four sections and a flip chart. The materials cover 14 subject headings at a sixth-grade reading level and are taught by a counselor. The counseling sessions were personalized to the individual needs and interests of the participants, with the counselor asking questions before each topic to determine their thoughts and adjust the discussion accordingly [18] (Table 1).

**Table1:** Counseling sessions

Sessions	Content	Time
First session (30–33 weeks)	Staying healthy during and after pregnancy Mother's knowledge about breastfeeding	60–90 min
Second session (34–36 weeks)	Preparation for labor and childbirth (labor and delivery, skin-to-skin contact, rooming-in, and feeding cues)	60–90 min
Third session (36–38 weeks)	The provided information covers a range of topics related to breastfeeding, including early and exclusive feeding, the advantages of breastfeeding, proper positioning and latching techniques, how to prepare for and maintain breastfeeding, and indicators of adequate expressed milk intake. Additionally, the information addresses potential issues that may arise during the first week of breastfeeding, returning to work while breastfeeding, preparing others to care for the baby, common concerns, and available support resources	60–90 min

Counseling was provided according to GATHER counseling principles in each session. The GATHER principles are “Greet”: warm and friendly communication and welcoming the woman. “Ask”: interaction with the woman and ask her about that session's topic. “Tell”: giving information and explanation about that session's topic. “Help”: Helping her to make the best choices. “Explain”: more explanation about the topic, and “Return”: follow up of the woman in the next sessions.

The content of the RSB program was examined and endorsed by five faculty members from the Hamadan School of Nursing and Midwifery. The control group received regular care, and both groups filled out the BSES questionnaire after the third session at the age of 38–36 weeks. Moreover, the researcher evaluated the mothers from both groups using the (Breastfeeding Assessment Tool) BBAT on the 3rd to 5th day post-delivery.

### Data collection tools

This study utilized various tools, such as demographic-clinical questionnaires, BSES, and BBAT. The demographic-clinical questionnaire consisted of questions about the woman's age, education, and occupation, as well as her spouse's age, education, and occupation. Additionally, the questionnaire included information on the body mass index, monthly income, gestational age upon entering the study, newborn gender, birth weight, type of birth, and gestational age at the time of delivery. To ensure the validity of the demographic questionnaire, the content validity method was employed, which involved soliciting feedback from ten Hamadan University of Medical Sciences members and incorporating their revised opinions.

The BSES was utilized to measure self-efficacy, consisting of 33 items graded on a Likert scale ranging from 1 to 5, indicating "I completely disagree" to "I completely agree," respectively [20]. A score between 33 to 76 indicated low self-efficacy, 77 to 121 indicated medium, and 121 to 165 indicated high self-efficacy [16]. The tool's reliability was established with a Cronbach alpha coefficient of 0.96 [20] in a previous study and 0.98 in the current study. The reliability of the BSES (0.82) was reported by Parsa et al. in Iran [21]. The BBAT measured breastfeeding performance on days 3 to 5 postpartum. This tool had four items, including "position," "attachment," "sucking," and "swallowing," each scored between 0 and 2 points on a 3-point Likert scale. Higher scores denoted more successful breastfeeding. The questionnaire's reliability was demonstrated with an alpha coefficient of 0.83 in a previous study [22] and a correlation coefficient of *r* = 0.86 in the current study, determined using the evaluation method by two assessors.

### Statistical analysis

Stata-13 software was used for data analysis. Central and dispersion indicators, frequencies, percentages, and tables described variables. The normal distribution of quantitative data was examined using the Kolmogorov–Smirnov test. Demographic variables between the two groups were compared by independent t-test, Mann–Whitney U test, Chi-square, and Fisher’s exact tests. The differences in self-efficacy scores and breastfeeding performance between the two groups were evaluated by the analysis of variance (ANCOVA). A significant level of *P* < 0.05 was considered in all statistical tests.

## Results

The study involved participants who were all homemakers with a mean age of 16.97(1.30) years. Upon entering the study, the mean gestational age of the samples was 32.45(1.04) weeks, and they were monitored until 3.53(0.76) days postpartum. The demographic and clinical characteristics of the two groups were relatively similar (*P* > 0.05). However, there was a significant difference in the mean neonatal weight between the groups, which was taken into account in the statistical analyses (Table 2).

**Table 2:** Characteristics of participants

Variable	Control (*n* = 30) Mean (SD)	Intervention (*n* = 30) Mean (SD)	*P*-value
Mother's age (year)	17.10 (1.29)	16.83 (1.31)	0.558^a^
Spouse's age (year)	26.50 (2.27)	25.90 (2.56)	0.341^b^
Gestational age (week)	32.23 (1.07)	32.67 (0.99)	0.110^b^
Gestational age at birth (week)	39.03 (0.92)	38.83 (1.17)	0.454^b^
Birth weight (gram)	3396 (375.235)	3162.50 (403.429)	0.024^b^
Mother’s education/n (%)			0.863^c^
Elementary	11(36.7)	13 (43.3)	
Middle school	16 (53.3)	15 (50.0)	
High school	3 (10.0)	2 (6.7)	
Spouse's education/n (%)			0.385^c^
Elementary	1 (3.3)	4 (13.3)	
Middle school	2 (6.7)	4 (13.3)	
High school	9 (30.0)	9 (30.0)	
Diploma and more	18 (60.0)	13 (43.3)	
Spouse's job			0.292^c^
Unemployed	1 (3.3)	3 (10.0)	
Employee	7 (23.3)	3 (10.0)	
Self-employed	22 (73.3)	24 (80.0)	
BMI			0.560^c^
Underweight	2 (6.7)	1 (3.3)	
Normal	24 (80.0)	27 (10.0)	
Overweight	4 (13.3)	2 (6.7)	
Economic Status			0.610^c^
Low	3 (10.0)	3 (10.0)	
Middle	11 (36.7)	15 (50.0)	
High	16 (53.3)	12 (40.0)	
Infant Gender			0.796^d^
Boy	15 (50.0)	16 (53.3)	
Girl	15 (50.0)	14 (46.7)	
Type of delivery			0.592^d^
Normal Vaginal Delivery	18 (60.0)	20 (66.7)	
Cesarean section	12 (40.0)	10 (33.3)	

^a^Mann-Whitney test

^b^Independent t-test

^c^Fisher's exact test

^d^Chi-square

The ANCOVA was used to comparing of self-efficacy and breastfeeding performance between two groups with controlling the effects of pre-intervention scores and newborns' weight. Results indicated a significantly lower mean score in BSE in the control group compared to the intervention group after intervention (*P* = 0.005), the mean difference (MD) = 16.01 (95% CI: 5.34,26.67), and the mean score of breastfeeding performance in the intervention group (6.30 ± 2.07), MD = 2.18 (95% CI: 1.11,3.24) was significantly higher than the control group (4.12 ± 2.07) (*P* = 0.001). Effect sizes were calculated for BSE and breastfeeding performance, with values of 0.77 (95% CI, 0.24,1.29) and 1.05 (95% CI, 0.5,1.5), respectively. These effect sizes suggest a substantial difference in self-efficacy and breastfeeding performance between the two groups (Table 3).

**Table 3:** Comparing of self-efficacy and breastfeeding performance between two groups

**Variable**	**Groups**	**Before intervention** **Mean(SD)**		**ANCOVA analysis results after the intervention**			
			**Adjusted** **mean (SD)**	**F**	***P*-Value**	**MD**^**a**^ **CI(0.95)**	**Cohen’s d** **CI(0.95)**
**Breastfeeding self-efficacy**	Control	94.03 (26.54)	100.02 (20.64)	8.42	0.005	16.01 (5.34,26.67)	0.77 (0.24,1.29)
	Intervention	109.67 (28.72)	116.03 (20.64)				
**Breastfeeding performance**	Control	-	4.12 (2.07)	15.86	0.001	2.18 (1.11,3.24)	1.05 (0.5,1.5)
	Intervention	-	6.30 (2.07)				

^a^Mean Difference

## Discussion

This study determined the effect of breastfeeding counseling with the RSB counseling approach on breastfeeding self-efficacy and performance in primiparous teenagers. The results indicated that counseling with the RSB approach increased BSE and improved breastfeeding performance in primiparous teenage mothers. Self-efficacy is a critical factor in exclusive breastfeeding and breastfeeding duration [19]. In this study, BSE in both groups was moderate before the intervention. Mofi et al. showed that breastfeeding self-efficacy was not optimal after childbirth [23]. Eslami et al. reported BSE was lower in younger mothers, and maternal age was a predictor of BSE, so that mothers aged under 19 years showed lower BSE [11]. Adolescents have a lower breastfeeding rate than older mothers [19]. Inexperience in breastfeeding and low education levels in primiparous teenage mothers are associated with lower self-efficacy [24]. Therefore, counseling support is more necessary for them [11].

In the present study, BSE and breastfeeding performance improved significantly after three counseling sessions in the third trimester of pregnancy. Aghababaei et al. claimed that individual counseling increased BSE in women aged 18–35 [16]. In their study four counseling sessions (one session at the end of pregnancy and three sessions after delivery) were held.

Shafaei et al. showed that prenatal group counseling could increase self-efficacy during breastfeeding and solve most breastfeeding problems in the postpartum period [15]. Although the exact time of intervention during pregnancy is not mentioned in their study, it shows the positive impact of counseling during pregnancy on BSE, the same as ours. Parsa et al. showed that self-efficacy and the continuity of exclusive breastfeeding increased in primiparous mothers in the fourth month of counseling with the GATHER model for four sessions during the first month after childbirth [21]. Compared to this study, the counseling sessions were held before birth in the present study. Prenatal counseling is advantageous as the mother is ready to start breastfeeding after childbirth, which can affect her breastfeeding consequences in the future.

Cantin et al. mentioned that a breastfeeding education program (about 13 training sessions) improves breastfeeding self-efficacy, knowledge, self-confidence, and satisfaction with teenagers in breastfeeding [19]. Self-efficacy increased significantly with three counseling sessions using the RBS approach in the present study. Therefore, it can be argued that BSE can be increased in teenagers with fewer sessions, and this result can be used for planning in this field.

Parry et al. showed that using the RSB counseling approach in group or individual prenatal counseling sessions improved breastfeeding tendencies and increased breastfeeding knowledge, early initiation of breastfeeding, and breastfeeding continuity and exclusivity [18]. In their study, counseling was done by a doctor, physician, resident physician, registered dietitian, nurse, and lactation professional; The consultation time was reportedly less than 15 min to more than 60 min, and the consultation was made only once in women aged over 18 years with a gestational age of 32 weeks. The RSB counseling approach was considered to be appropriate by all participants, and 88% considered the intervention time to be appropriate at that gestational age. The less time allocation to counseling in their study might be because of older participants' age. Javorski et al. reported that using a flip chart as an educational tool positively affected BSE and maintaining exclusive breastfeeding [25]. In our study, using flip charts also influenced breastfeeding counseling for teenagers.

Bhat et al. showed that 59% of mothers used the wrong breastfeeding way, indicating the need for a counseling support system to improve breastfeeding performance [26]. Lack of breastfeeding education and counseling during pregnancy causes early cessation of breastfeeding. Increasing mothers' knowledge of breastfeeding improves their performance [27]. Parsa et al. evidenced a positive effect of postpartum breastfeeding counseling on the mother's breastfeeding performance [17]. In their study, four breastfeeding counseling sessions after delivery could improve breastfeeding performance in the 4th month after delivery. While in the present study, prenatal counseling led to improved performance in the first days after childbirth, which are critical days in terms of the initiation and continuation of breastfeeding.Javorski et al. showed an educational intervention using a flip chart in the second, fourth, and eighth weeks after birth positively influenced maintaining exclusive breastfeeding [25]. It can be concluded that the use of educational tools both before and after childbirth can positively affect breastfeeding performance.

One of the limitations of this research was the carelessness of participants during counseling due to their young age, low literacy level, and unfamiliarity with breastfeeding. So, the authors tried to overcome this limitation by allocating more time, using more straightforward language, flip charts, and providing educational pamphlets in simple language. Another limitation of this study is that the intervention's impact on infant outcomes, including the proportion of exclusive breastfeeding, the timing of the first feed, weight trends, and post-discharge feeding patterns, weren’t measured. An advantage of this study is the research on teenagers, who have not been sufficiently studied to improve their breastfeeding performance despite their poor breastfeeding consequences. Another benefit is the intervention before giving birth, which helps the woman to start breastfeeding with more self-efficacy and to have a more appropriate performance from early breastfeeding. Moreover, using the GATHER method and the RSB model in counseling teenagers showed that a comprehensive approach to education could effectively promote BSE in them. It was tried to increase the generalizability of the results by providing details on the samples, the location and the method.

## Conclusions

The study showed that counseling with the RSB approach during the prenatal period increased the level of BSE and improved breastfeeding performance among nulliparous teenagers. As a result, it is advisable to create a customized counseling program for teenagers during pregnancy to help them begin breastfeeding with higher self-efficacy and improved performance.

## Data Availability

The datasets used during the current study are available from the corresponding author on reasonable request.

## Abbreviations

RSB: Ready Set BABY
EBF: Exclusive breastfeeding
BSE: Breastfeeding self-efficacy
BSES: Breastfeeding self-efficacy Scale
BBAT: Bristol Breastfeeding Assessment Tool
